# On the Microstructure and Isothermal Oxidation of the Si-22Fe-12Cr-12Al-10Ti-5Nb (at.%) Alloy

**DOI:** 10.3390/ma12111806

**Published:** 2019-06-03

**Authors:** Ofelia Hernández-Negrete, Panos Tsakiropoulos

**Affiliations:** Department of Materials Science and Engineering, Sir Robert Hadfield Building, The University of Sheffield, Mappin Street, Sheffield S1 3JD, UK; ochernandeznegrete@gmail.com

**Keywords:** coatings, intermetallics, silicides, pest oxidation, high temperature oxidation, Nb-silicide based alloys

## Abstract

Nb-silicide based alloys are new ultra-high temperature materials that could replace Ni-based superalloys. Environmentally resistant coating system (s) with αAl_2_O_3_ or SiO_2_ forming bond coat alloys that are chemically compatible with the Nb-silicide based alloy substrates are needed. This paper makes a contribution to the search for non-pesting bond coat alloys. The microstructure and isothermal oxidation at 800 °C of the silicide-based alloy Si-22Fe-12Cr-12Al-10Ti-5Nb (OHC2) were studied. The cast alloy exhibited macrosegregation of all elements. The microstructures in the cast alloy and after the heat treatment at 800 °C consisted of the same phases, namely TM_6_Si_5_, TM_5_Si_3_ (TM = transition metal), FeSi_2_Ti, Fe_3_Al_2_Si_3_, (Fe,Cr)(Si,Al), and an unknown phase of dark contrast. The latter two phases were not stable at 950 °C, where the TMSi_2_ was formed. There was evidence of endothermic reaction(s) below 1200 °C and liquation at 1200 °C. The alloy followed parabolic oxidation kinetics after the first hour of isothermal oxidation at 800 °C, did not pest, and formed a self-healing scale, in which the dominant oxide was Al_2_O_3_. The alloy was compared with other alumina or silica scale-forming intermetallic alloys and approaches to the design of bond coat alloys were suggested.

## 1. Introduction

Currently, Ni-based superalloys are the structural materials of choice for the aerofoils used in the hottest parts of gas turbine engines. Superalloy aerofoils are operated with environmentally resistant coatings and internal cooling, but even with these features their high temperature capability has reached a limit owing to the melting temperatures of Ni and Ni-based superalloys [1,2]. The aerospace industry needs power systems with better efficiency, which is achievable with increase of the turbine entry temperature. As the latter increases beyond 1500 °C, new high temperature materials will be required. These materials should have some inherent resistance to high temperature oxidation and good mechanical properties at room, intermediate, and high temperatures.

Nb-silicide-based alloys (also known as Nb-silicide in-situ composites) can offer a balance of properties [3]. A major challenge for the development of Nb-silicide-based alloys is their oxidation, which can change from catastrophic at intermediate temperatures (pest oxidation) to complex at high temperatures [3]. Even though the oxidation has been dramatically improved without compromising their ductility, Nb-silicide based alloys do not form a protective scale because the concentrations of the key elements Al, Cr, Si, and Ti are restricted by property and melting temperature requirements.

The Nb-silicide-based alloys would require environmentally resistant coatings, such as the Ni-based superalloys [1,2,4]. It is anticipated that Nb-silicide-based alloy (substrate) surface temperatures could be up to 150 degrees higher compared with state of the art internally cooled and coated single crystal Ni-based superalloy aerofoils. This will only be possible using a materials system approach, where the latter will comprise the substrate, which should have some inherent oxidation resistance, and an environmentally resistant coating system. The latter could consist of bond coating and a ceramic thermal barrier. In other words, an environmentally resistant coating for Nb-silicide based alloys could have a structure similar to that of the coatings used for Ni-based superalloys, namely an oxidation resistance bond coat (BC), thermally grown oxide (TGO), and ceramic top coat [5,6]. The TGO formed in situ in coated Ni-based superalloys is αAl_2_O_3_. Research must find BCs that are compatible with Nb-silicide based alloys that could form αAl_2_O_3_ (preferred) or SiO_2_ TGO. This has been discussed in recent publications by our group [7,8,9].

It has been suggested that an approach could be to develop BCs based on Si-Cr-Fe alloys with oxidation resistant phases and limited oxygen penetration [10]. Silicides and Si-rich intermetallic alloys, such as the R512 coatings [11], can form SiO_2_ coatings with the potential to form a glass layer able to prevent oxidation, scale cracking, and spallation. SiO_2_ would also prevent the increase of the substrate surface temperature and can be self-healing [12]. However, even though complex Si-rich intermetallics were found to provide optimum protection against oxidation to conventional Nb-rich alloys (i.e., not Nb-silicide based alloys), poor mechanical behavior has been reported for coatings with high silicon contents that formed SiO_2_ protective scales [13,14].

Iron-modified silicide coatings have provided oxidation protection to conventional Nb-rich alloys. This was attributed to the selective oxidation of silicon in coatings with FeSi, FeSi_2_, FeSi_2_Ti, and TM_7_Si_6_ phases in their microstructure, where TM = Fe,Ti,Cr,Nb [10,15,16]. Data about the oxidation of modified (alloyed) iron silicides is scarce. Additions of the above elements could alter the melting point of Fe silicides and affect their oxidation and mechanical performance at high temperatures. It is not known whether formation of protective oxides, such as Cr_2_O_3_, SiO_2_, and Al_2_O_3_, could be possible with Al addition to Fe-based silicide coatings.

Studies of the oxidation of Si-rich Fe-Si alloys at intermediate and high temperatures attributed a reduction in the oxidation rate to the formation of SiO_2_ [17]. Amorphous SiO_2_ has been the desirable oxidation product for these alloys because it has good adhesion to silicide phases, low oxygen diffusion, and ability to resist plastic deformation at high temperatures.

For Nb-silicide base alloys, different coating systems have been considered to promote the formation of alumina or silica scales [18,19,20]. Different configurations of coating systems for turbine blades of Nb-silicide based alloys have been proposed [21,22]. For example, the coating could consist of a diffusion barrier layer and inert bond coat, or in addition to these could also have a platinum group metal layer, a Cr_2_Nb Laves layer, and a chromium layer. These would form a bond coat, on top of which a ceramic top coat (thermal barrier) would be deposited.

One approach in the design of an environmentally resistant coating system for a Nb-silicide based alloy is to use a layered multi-material or functionally gradient BC with decreased Nb content and increased capability to form Si and Al oxides from the Nb-silicide-based substrate surface towards the top coat. The latter would be deposited on the top component of the BC (i.e., the one furthest away from the substrate) that could form in situ αAl_2_O_3_ (preferred) or SiO_2_ TGO. Recently, our group reported on research seeking suitable BC alloys [7,8,9]. The motivation of the research reported in this paper was to find out the effect of Al addition on the microstructure and oxidation of silicide based coatings of the Si-Fe-Cr-Ti-Al-Nb system. In this paper we report on an alloy of this system.

The structure of the paper is as follows. First we discuss the selection of the composition of the alloy to be studied. Then, the experimental details are given followed by the results about the cast and heat treated microstructures and the isothermal oxidation of the alloy. In the discussion we first deliberate on the microstructure of the alloy and then on its oxidation. The alloy is compared with other alumina or silica scale-forming alloys and alloys are suggested for BCs.

## 2. Selection of Alloy Composition

The alloying behavior of the key phases in Nb-silicide based alloys can be described using the parameters δ (related to atomic size), Δχ (related to electronegativity), and the number of valence electrons per atom filled into the valence band (VEC) [23,24,25]. These three parameters are also key in the alloy design methodology NICE (Niobium Intermetallic Composite Elaboration) that has been developed for the design and selection of new Nb-silicide-based alloys with a balance of mechanical and oxidation properties [3]. NICE was used to select the alloys MG5, MG6, and MG7 [7,8] (see below).

Recently, in maps of the parameters δ, Δχ, and VEC (Figure 23 in a previous study [9]), it was shown that two alloys with microstructures consisting of Si-rich transition metal silicides, namely the alloys OHC1 and OHC5 with nominal compositions (at.%) Si-23Fe-15Cr-15Ti-1Nb (OHC1) and Si-25Nb-5Al-5Cr-5Ti (OHC5), correlated well with Nb-Ti-Si-Al-Hf alloys with microstructures consisting of Si-rich transition metal silicides and transition metal aluminides, namely the alloys MG5, MG6, and MG7 of nominal compositions (at.%) 14.5Nb-27Si-22.5Ti-32.5Al-3.5Hf (MG5), 13.5Nb-23Si-23Ti-37Al-3.5Hf (MG6), and 13Nb-24Si-24Ti-35Al-4Hf (MG7), respectively. The alloy OHC5 was “closer” to the latter three Al-rich alloys (32.5 < Al < 37 at.%), and similar to them it formed alumina scale but at a significantly lower Al concentration (5 at.%). The alloy OHC1, with no Al addition, was “further away” and formed scales composed of silica, chromia, and titania oxides. In a previous study [9] it was suggested that the alloy OHC1 would not be suitable as a BC component for Nb-silicide based components owing to the likelihood of melting above 1100 °C. Would the same be the case if Al were to be present in the alloy, or, to put it in another way, what would the effect of Al addition be on microstructure stability and oxidation behavior of an OHC1 type alloy?

To answer this question, we selected a silicide alloy based on the alloy OHC1 that was designed to have Al substituting Cr, Si, and Ti, and its microstructure to include the same silicides as OHC1, but (a) with the constraint the alloy to be located between the alloys OHC1 and OHC5 in the aforementioned maps and (b) with three requirements, namely the alloy (i) to have no stable Nb solid solution in its microstructure, (ii) to not pest, and (iii) to not form Nb oxides. Considering the phase equilibria for the ternary systems Cr-Si-Ti [26], Fe-Si-Ti [27], Cr-Fe-Si [28], Cr-Nb-Si [29] and Al-Fe-Si [30], the composition of the alloy OHC1 [9] and (a) and (b), the Si and Al concentrations, respectively, of 40 and 12 at.% were selected to penetrate the alloy microstructures consisting of TM_6_Si_5_ (TM = transition metal), TM_5_Si_3_, B20, and C40 compounds. The Al addition was expected to promote the formation of the Fe_3_Al_2_Si_3_ phase. The reasons for the choice of above phases were discussed in a previous study [9].

**Figure 1:**
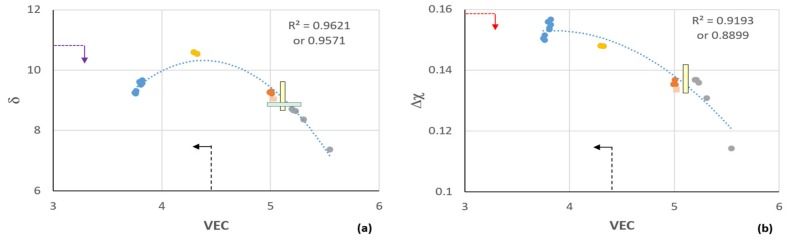

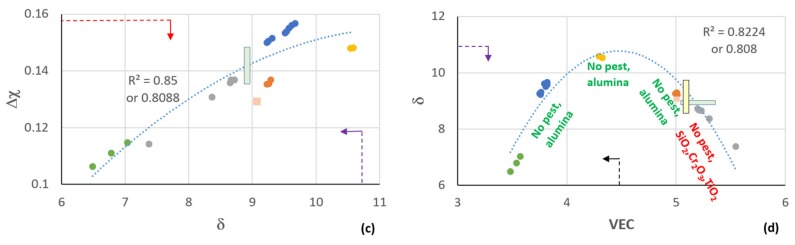
Maps of the parameters δ, Δχ, and VEC for the alloys OHC2 (orange), OHC1 (grey), OHC5 (gold), MG5, MG6, MG7 (blue), and Zone A in alloy MG7 (green): (**a**) δ versus VEC, (**b**) Δχ versus VEC, (**c**) Δχ versus δ, (**d**) δ versus VEC. Data points for Zone A in OHC2 are shown by squares. For the yellow and green boxes and dashed lines and arrows, see text. (**a**–**d**) The lower *R*^2^ value is for data that includes Zone A of the alloy OHC2.

The nominal composition (at.%) of the alloy was Si-22Fe-12Cr-12Al-10Ti-5Nb (OHC2). The Figure 1a,b shows the alloys OHC1, OHC2, and OHC5, and MG5, MG6, and MG7 [7,8] without the data for Zone A of the alloy MG7 [7], which is included in the Figure 1c,d. The correlations are good. When the data for Zone A was included, the R^2^ values decreased in the δ versus VEC map (Figure 1d) and Δχ versus VEC map (not shown, R^2^ = 0.5504) and increased in the Δχ versus δ map from R^2^ = 0.7153 (map not shown) to R^2^ = 0.85 (Figure 1c).

As was the case in previous studies [7,8,9], the alloy was not studied as a coating applied on a Nb-silicide-based substrate in order to eliminate the effects of substrate and coating process on microstructure and oxidation.

## 3. Experimental

We used arc melting with a non-consumable tungsten electrode and a water-cooled copper crucible, a voltage of 50 V, and a current of 650 A to prepare the alloy from pure elements (≥99.9 wt.% purity) in a Ti-gettered Ar atmosphere. The button was melted 5 times to homogenize its composition. For the heat treatments we used an alumina tube furnace and a Ti-gettered Ar atmosphere. The specimens were polished, wrapped in Ta foil to minimize contamination by oxygen, and were placed in an alumina crucible.

The microstructures were characterized using Scanning Electron Microscopy (SEM) and X-ray diffraction (XRD). Philips PSEM 500 SEM (Philips-ThermoFisher Scientific, Hillsboro, OR, USA), Jeol JSM 6400 SEM (Jeol, Tokyo, Japan), and Inspect F FEG SEM microscopes (ThermoFisher Scientific, Hillsboro, OR, USA) were used. The back scatter electron (BSE) mode was used to study the microstructures with qualitative and quantitative EDS analysis (Oxford Instruments, Abington, UK) of the alloy and phases (20 kV for EDS quantitative analysis and 20 kV and 15 kV, respectively, for the X-ray elemental maps of the scale surface and cross sections of oxidized specimens). EDS standardization was performed using specimens of high purity Nb, Ti, Cr, Fe, Si, Al, and Co standards that were polished to 1 μm finish. The EDS was calibrated prior to analysis with the Co standard. At least five large area analyses were performed in the top, bulk, and bottom of the button, and at least ten analyses were obtained from each phase with size ≥5 μm to determine actual compositions.

A Siemens D500 XRD diffractometer (XRD, Hiltonbrooks Ltd, Crew, UK) with CuKα radiation (λ = 1.540562 Å), 2θ from 20°–120°, and a step size of 0.02° was used. For glancing angle XRD (GXRD), a Siemens D5000 diffractometer with Cu Kα1 and Kα2 radiation (λ = 1.54178 Å), 2θ from 10°–100°, and a step size of 0.02° was used. Peaks in the XRD diffractograms were identified by correlating data from the experiments with that from the JCPDS data (International Centre for Diffraction Data). The scan type used for GXRD was a detector scan, while for regular specimens it was a locked coupled scan. Prior to GXRD experiments the glancing angle was selected with the aid of the AbsorbDX software (Bruker, Karlsruhe, Germany), which evaluates the X-ray penetration depth for particular glancing angle conditions.

The isothermal oxidation was studied at 800 °C for 100 h in laboratory air using thermo-gravimetric analysis (TGA) in a Netzsch STA F3 TG/DSC analyzer (Netzsch Gmbh, Waldkraiburg, Germany) with a SiC furnace and alumina crucible specimen holder with air flow rate of 20 mL/min and with heating and cooling rates of 3 °C/min. Cubic specimens of size 3 mm × 3 mm × 3 mm were used for the TGA experiments. The specimens were cut from the as-cast alloy and polished to 800 grit SiC finish. For the DSC experiments a Rh/Pt furnace was used in the Netzsch STA F3 TG/DSC analyzer with an Ar flow rate of 20 mL/min. The specimens for thermal analysis and isothermal oxidation were selected from the bulk of the cast button.

## 4. Results

### 4.1. Cast Alloy

**Figure 2:**
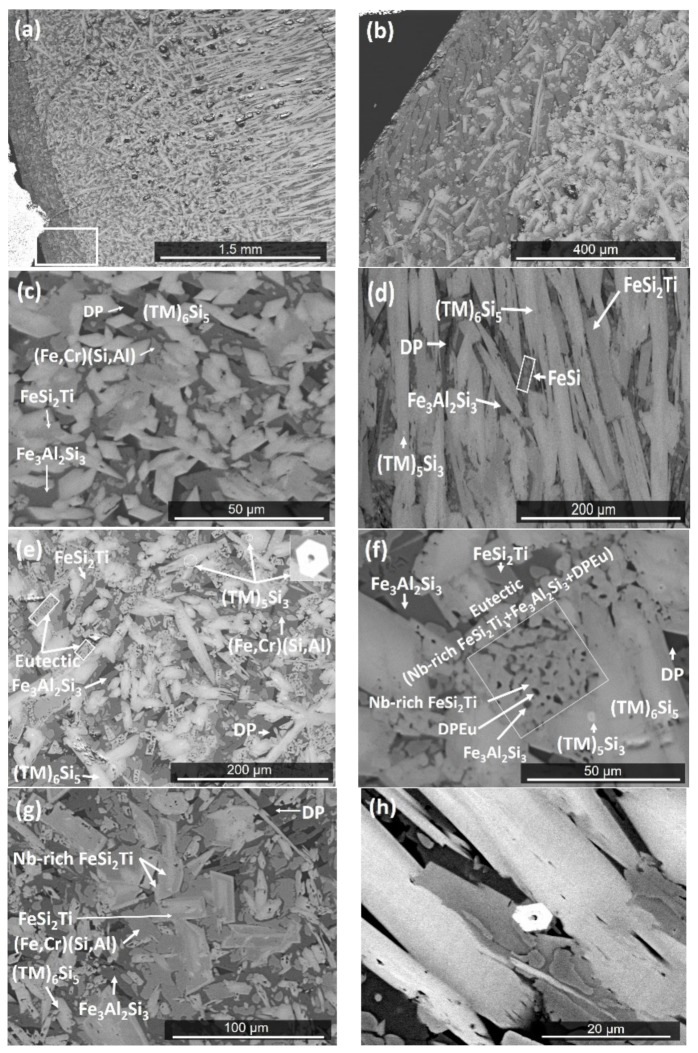
BSE images of OHC2-AC: (**a**) Zone A formed near water cooled copper crucible (×40), (**b**) microstructure of area indicated by insert in (a), (**c**) top, (**d**) bulk, (**e**) near bottom, (**f**) near bottom showing eutectic, (**g**) Zone A, and (**h**) bulk showing hexagonal grains of TM_5_Si_3_.

The actual composition (at.%) of the cast alloy (OHC2-AC) was Si-21.5Fe-13.7Al-11.2Cr-8.5Ti-4.9Nb. This was the average composition of the large area analyses from all parts of the button. There was macrosegregation of all the elements. The bottom of the button was leaner in Nb, Ti, Cr, and Si and richer in Fe and Al compared with its other parts. Near the copper crucible wall a zone was formed (Figure 2a,b) with average composition Si-27.9Fe-25.5Al-7.1Cr-3Ti-1.1Nb. We shall refer to this as Zone A [7]. The microstructures are shown in Figure 2c–h.

**Figure 3:**
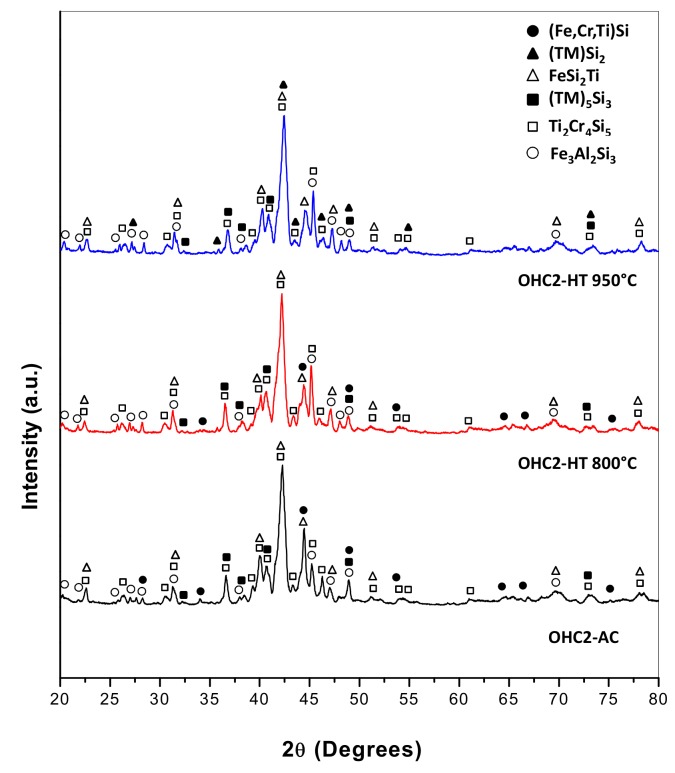
X-ray diffractograms of the cast and heat treated alloy OHC2.

The EDS (Table 1) and XRD (Figure 3) data confirmed that the microstructure consisted of six phases, namely the TM_6_Si_5_ silicide with light graded contrast, the TM_5_Si_3_ silicide that exhibited white contrast (see Figure 2d,h) (TM = Nb, Ti, Fe, Cr), the phases τ_1_ = FeSi_2_Ti and (Fe,Cr)(Si,Al) with similar grey contrast but different morphology, the Fe_3_Al_2_Si_3_ phase with the dark grey contrast, and the dark phase (DP) with dark contrast in Figure 2. The average compositions of these phases are given in Table 1. The (Fe,Cr)(Si,Al), Fe_3_Al_2_Si_3_, and dark phase (DP) essentially were Nb- and Ti-free.

The TM_6_Si_5_ silicide was orthorhombic with the V_6_Si_5_ as prototype and Ibam space group (JCPDS card 54-381). The TM_5_Si_3_ silicide was based on the Ti_5_Si_3_ hexagonal silicide with the P6_3_/mcm space group (JCPDS card 89-3721). The FeSi_2_Ti phase had the MnSi_2_Ti prototype with the Pbam space group (JCPDS card 75-2180). Its composition matched with the composition of the τ_1_ phase in [31]. The (Fe,Cr)(Si,Al) had the B20 structure with the P2_1_3 space group (JCPDS card 38-1397). The Fe_3_Al_2_Si_3_ phase was triclinic with the P-1 space group (JCPDS card 87-1920).

The microstructure in the top of the button is shown in Figure 2c. The TM_6_Si_5_ silicide had a facetted morphology and microsegregation. The FeSi_2_Ti had an irregular shape and was mainly formed at the grain boundaries of the TM_6_Si_5_, while the Fe_3_Al_2_Si_3_ exhibited facetted plate like morphology. Small hexagonal grains of the TM_5_Si_3_ silicide of bright contrast were randomly distributed and formed at a low volume fraction only in those areas of the top that were close to the bulk. The volume fraction of the dark phase (DP) also was very low.

**Figure 4:**
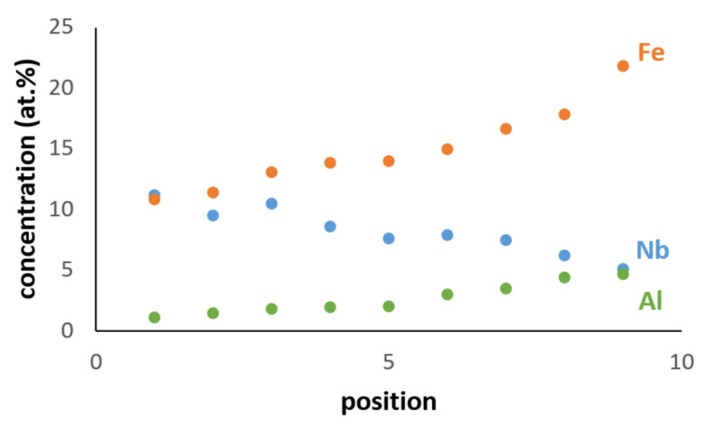
Al, Fe, and Nb concentrations from near the bulk (center) of one TM_6_Si_5_ grain to near its edge. Center and edge correspond to positions zero and ten, respectively.

In the bulk of the button, the microstructure comprised of a large volume fraction of facetted TM_6_Si_5_. There was microsegregation in this phase that resulted in graded BSE contrasts (Figure 2d), showing a light contrast in the center and dark grey contrast in the edges. The center of TM_6_Si_5_ was richer in Nb, Ti, Si, and Cr, and the edges were rich in Fe and Al (Figure 4). For example, in one TM_6_Si_5_ grain the concentrations (at.%) of these elements were about 11.2Nb, 10.9Fe, 18.8Cr, 12.4Ti, 44.8Si, and 1.9Al in the bulk, and 5.2Nb, 21.9Fe, 14.1Cr, 11.4Ti, 42.7Si, and 4.7Al at the edge of the grain. Similar variations in composition were observed in other TM_6_Si_5_ grains. In general, the change of the concentrations of Cr, Si, and Ti from the bulk to the edge of TM_6_Si_5_ grains was not as striking as for Fe and Nb (Figure 4). The FeSi_2_Ti and (Fe,Cr)(Si,Al) phases had similar grey contrast and different morphology. The FeSi_2_Ti was facetted (like rectangles) and formed on the grain boundaries of the TM_6_Si_5_. The (Fe,Cr)(Si,Al) was mostly found as isolated particles in the vicinity of FeSi_2_Ti. The Fe_3_Al_2_Si_3_ was cracked. The dark phase (DP) was mainly found between the TM_6_Si_5_ and the Fe_3_Al_2_Si_3_, was formed at a low volume fraction, and its Si and Al concentrations varied significantly. The contrast of the small hexagonal grains of TM_5_Si_3_ was darker in the bulk and the silicide was distributed unevenly.

Near the bottom of the button (Figure 2e), the volume fraction of the TM_6_Si_5_ was reduced. The TM_5_Si_3_, Fe_3_Al_2_Si_3_, and DP phases were present with the same features as in the bulk of the button. In this area the TM_6_Si_5_ had more voids and cracks. A ternary eutectic was formed. This eutectic consisted of the FeSi_2_Ti phase (light contrast), the Fe_3_Al_2_Si_3_ phase with dark grey contrast and a dark contrast phase (labelled DPEu in the Figure 2f), with average compositions given in Table 1. The (Fe,Cr)(Si,Al) was in the form of isolated particles embedded in the Fe_3_Al_2_Si_3_ phase. The EDS data for Fe_3_Al_2_Si_3_ and DPEu could be not very accurate because very small areas were analyzed. However, it was clear that the phase with the dark grey contrast was the Fe_3_Al_2_Si_3_.

In Zone A the volume fraction of the dark phase (DP) was slightly higher than in the rest of the button (Figure 2g). The TM_6_Si_5_, FeSi_2_Ti, and (Fe,Cr)(Si,Al) phases were present at lower volume fraction and the volume fraction of the Fe_3_Al_2_Si_3_ was the same as in the rest of the button. The FeSi_2_Ti was facetted (like trapezoids) and exhibited microsegregation; its brighter and darker contrast areas, respectively, were Nb-rich and Nb-poor. The (Fe,Cr)(Si,Al) surrounded these facetted grains (Figure 2g). The Fe-rich TM_6_Si_5_ was either facetted or had irregular shape. The TM_5_Si_3_ and eutectic were not observed in the Zone A.

**Figure 5:**
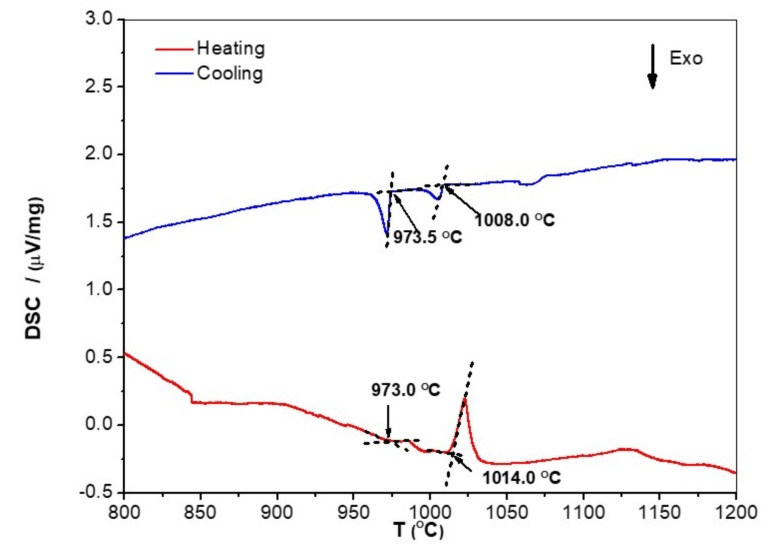
DSC trace of the alloy OHC2.

In the DSC trace (Figure 5) there was a small endothermic peak at about 973 °C, with a corresponding strong peak on cooling. Krendelsberger et al. [32] gave 975 °C as the melting temperature of Fe_3_Al_2_Si_3_. The second peak on heating was very strong and could correspond to an invariant reaction. This peak could be related to the ternary eutectic L → FeSi_2_Ti + Fe_3_Al_2_Si_3_ + DPEu. There was also a broad endothermic peak at about 1125 °C but with no corresponding peak on cooling. The microstructure of the alloy was studied after heat treatments at 800, 950, and 1200 °C for 48 h.

### 4.2. Heat Treated Alloy

**Figure 6:**
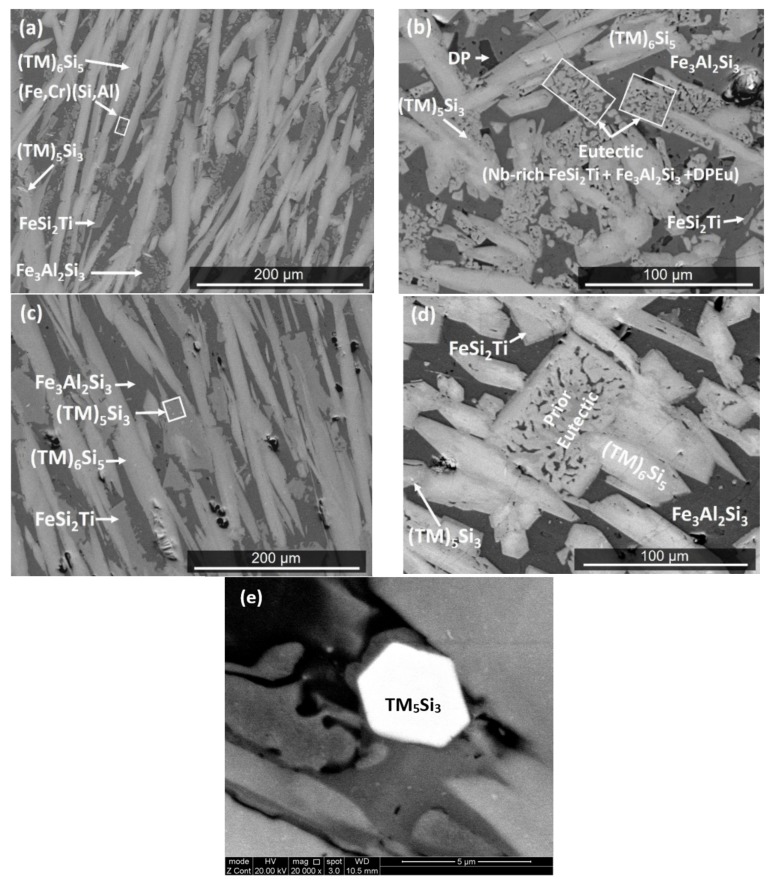
BSE images of the microstructures of the heat treated alloy, (**a**,**b**) OHC2-HTA, (**c**,**d**) OHC2-HTB, and (**e**) high magnification image of hexagonal TM_5_Si_3_ particles in (**d**).

The actual composition after the heat treatment at 800 °C (OHC2-HTA) was Si-21.5Fe-11.4Cr-12.9Al-8.6Ti-5Nb, essentially the same as the cast alloy. Chemical inhomogeneity and Zone A were still present. The latter was richer in Al compared with OHC2-AC (Si-27.1Fe-29.3Al-6.6Cr-2.3Ti-0.8Nb). According to the XRD (Figure 3) and EDS data, the microstructure consisted of the same six phases, namely the TM_6_Si_5_, TM_5_Si_3_, FeSi_2_Ti, (Fe,Cr)(Si,Al), Fe_3_Al_2_Si_3_, and the dark phase (DP). The compositions of the phases essentially were the same as in the cast alloy, with the exception of the dark phase (DP), which was richer in Al and poorer in Si (3.5(0.7)Cr-22.6(0.8)Fe-59.1(2.3)Al-14.3(1.4)Si-0.5Ti-0.1Nb, in parentheses are given the standard deviations). The typical microstructures of OHC2-HTA are shown in Figure 6a,b.

It can be seen that the microstructure had become coarser. There was some homogenization of the TM_6_Si_5_ silicide, the FeSi_2_Ti appeared to be more facetted, and although it was mainly found in the boundaries of the TM_6_Si_5_, it was also found as isolated plates. The volume fraction of Fe_3_Al_2_Si_3_ had increased. The dark phase (DP) was not found in the top and bulk of the button but was still present near the bottom, where the retained prior ternary eutectic was observed (Figure 6b) with average composition essentially the same as in the cast alloy. In Zone A, the volume fraction of TM_6_Si_5_ was reduced and its chemical inhomogeneity was still present. Tiny hexagonal particles of the TM_5_Si_3_ with very bright contrast, Fe_3_Al_2_Si_3_ plates, and the dark phase (DP) were observed.

The actual composition after the heat treatment at 950 °C (OHC2-HTB) was Si-21.5Fe-11.5Cr-13Al-9.9Ti-4.3Nb, essentially the same as the cast alloy. The typical microstructure in the bulk of OHC2-HTB is shown in Figure 6c. According to the XRD (Figure 3) and EDS data, the microstructure consisted of the TM_6_Si_5_, FeSi_2_Ti, Fe_3_Al_2_Si_3_, TM_5_Si_3_, and TMSi_2_ phases. The (Fe,Cr)(Si,Al) and dark phase (DP) were not present and the Cr-rich TMSi_2_ silicide was the new compound formed at this temperature. The chemical compositions of TM_5_Si_3_ and Fe_3_Al_2_Si_3_ essentially were the same as in the cast alloy, the TM_6_Si_5_ was richer in Ti and poorer in Fe (8.2Nb-14.7Ti-19.1Cr-11.9Fe-1.7Al-44.4Si), the FeSi_2_Ti was richer in Ti and poorer in Al (4.3Nb-17Ti-4.6Cr-23.1Fe-10.5Al-40.5Si) and the average composition of TMSi_2_ was 1.1Nb-2.9Ti-22.9Cr-7.4Fe-18.6Al-47.1Si.

The microstructures in the top and bulk were similar (Figure 6c). The FeSi_2_Ti had become coarser, was observed at the grain boundaries of TM_6_Si_5_, and sometimes linked TM_6_Si_5_ grains. Tiny hexagonal particles of TM_5_Si_3_ with very bright contrast were present. Near the bottom the areas of prior eutectic consisted of two phases, namely the Nb-rich FeSi_2_Ti and Fe_3_Al_2_Si_3_ phases. The DPEu was absent (Figure 6d). These prior eutectic areas were now richer in Ti and poorer in Al (5.9Nb-13.8Ti-5.6Cr-21.9Fe-12.4Al-40.3Si), with Si + Al = 52.7 at.% compared with 55.2 at.% in OHC2-AC. In the bottom of the button the same microstructure was observed, plus the TMSi_2_ silicide.

When the alloy was heat treated at 1200 °C the specimen had “collapsed”, owing to liquation. The microstructure was similar to that of the cast alloy.

### 4.3. Oxidation

We used the equation $ln\left(\Delta w\right)=lnK+nlnt$ for the analysis of the TGA data. In this equation $\Delta w=\frac{\Delta m}{A}$, where Δ*w* is the weight change per unit area, Δ*m* is the weight change, and *A* is the surface area before exposure, and K and *t* respectively are the reaction rate constant and the exposure time. For the linear, parabolic, and sub-parabolic or cubic oxidation kinetics, the values of n are 1, 0.5, and ≤0.3, respectively. When there was more than one mechanism involved, the corresponding section in the TGA data was used to determine the oxidation kinetics using the equations $\Delta {w=k}_{l}\cdot t$ for linear oxidation and ${\left(\Delta w\right)}^{2}{=k}_{p}\cdot t$ for parabolic oxidation, where k_l_ and k_p_, respectively, are the linear and parabolic rate constants [33].

**Figure 7:**
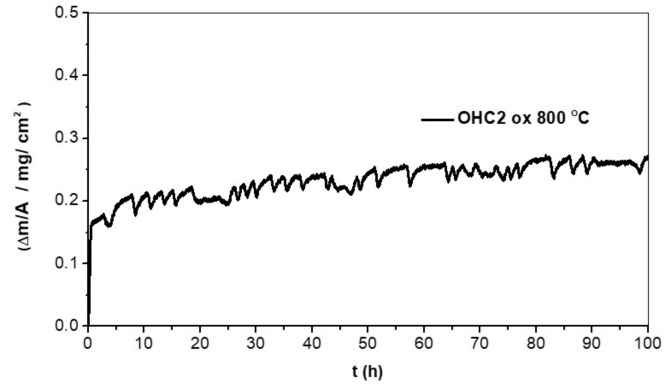
TGA data for isothermal oxidation in air at 800 °C for 100 h.

The TGA data for the isothermal oxidation in air at 800 °C for 100 h is shown in the Figure 7. The alloy did not pest and gained weight per unit area of 0.27 mg/cm^2^. Its n value was 0.14 and its oxidation behavior was sub-parabolic. There was a very short initial period (1 h) of oxidation, in which the alloy gained weight with linear kinetics (k_l_ = 1.03 × 10^−7^ g/cm^2^s), followed by a long period (99 h) of parabolic oxidation with k_p_ = 1 × 10^−13^ g^2^/cm^4^s. The cubic specimen had retained its shape and had well-defined edges; it had a black color with some blue and reddish tones. The scale remained attached and there was no evidence of scale spallation.

**Figure 8:**
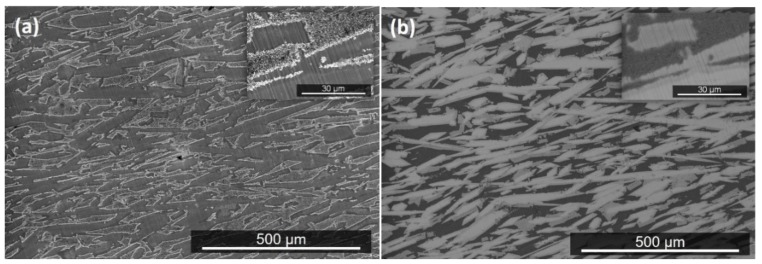
Scale of the alloy OHC2 after isothermal oxidation at 800 °C for 100 h: (**a**) SE image, (**b**) BSE image.

Figure 8a shows the morphology of the surface of the scale. A continuous and adherent bright oxide with darker contrast was formed over the Fe-rich areas of TM_6_Si_5_ grains (Figure 8b). Similar characteristics were observed for the oxide formed over the FeSi_2_Ti phase. Blade-like whiskers grew over the Fe_3_Al_2_Si_3_ (Figure 8a). The thickness of the scale varied between 1 and 2 μm.

**Figure 9:**
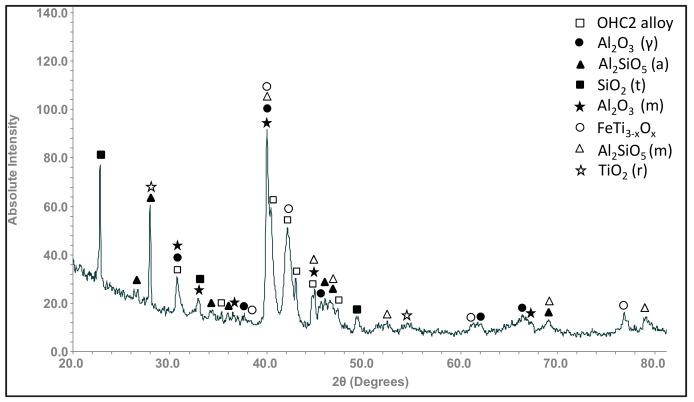
GXRD data (θ = 5°) for the scale formed on the alloy OHC2 at 800 °C for 100 h.

**Figure 10:**
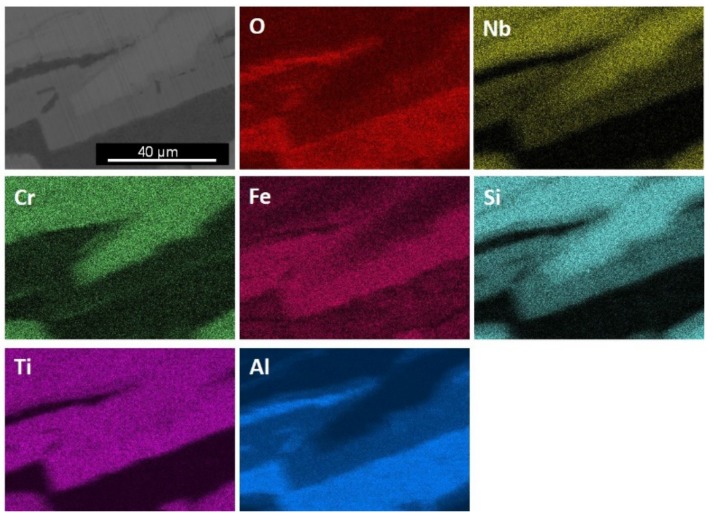
X-ray elemental maps of scale surface after isothermal oxidation at 800 °C for 100 h, BSE image.

The GXRD data had peaks that corresponded to the cubic γAl_2_O_3_ (JCPDS 29-63) and monoclinic θAl_2_O_3_, labelled Al_2_O_3_ (m) in Figure 9 (JCPDS 35-121), monoclinic Al_2_SiO_5_ (JCPDS 44-27) and anorthic Al_2_SiO_5_, labelled Al_2_SiO_5_ (a) (JCPDS 11-46), SiO_2_ trydimite (JCPDS 82-1556), labelled SiO_2_ (t), FeTi_3−*x*_O*_x_* (JCPDS 09-320), and tetragonal TiO_2_, rutile (JCPDS 82-514). The X-ray elemental maps of the scale surface showed that Al_2_O_3_ formed over Fe_3_Al_2_Si_3_ and that over the FeSi_2_Ti formed the Al_2_SiO_5_ and Ti*_x_*Fe_(3−*x*)_O*_x_* (Figure 10). Over the TM_6_Si_5_ formed SiO_2_, Al_2_O_3_, and some (Ti,Cr,Nb)O_2_ rutile type oxide.

**Figure 11:**
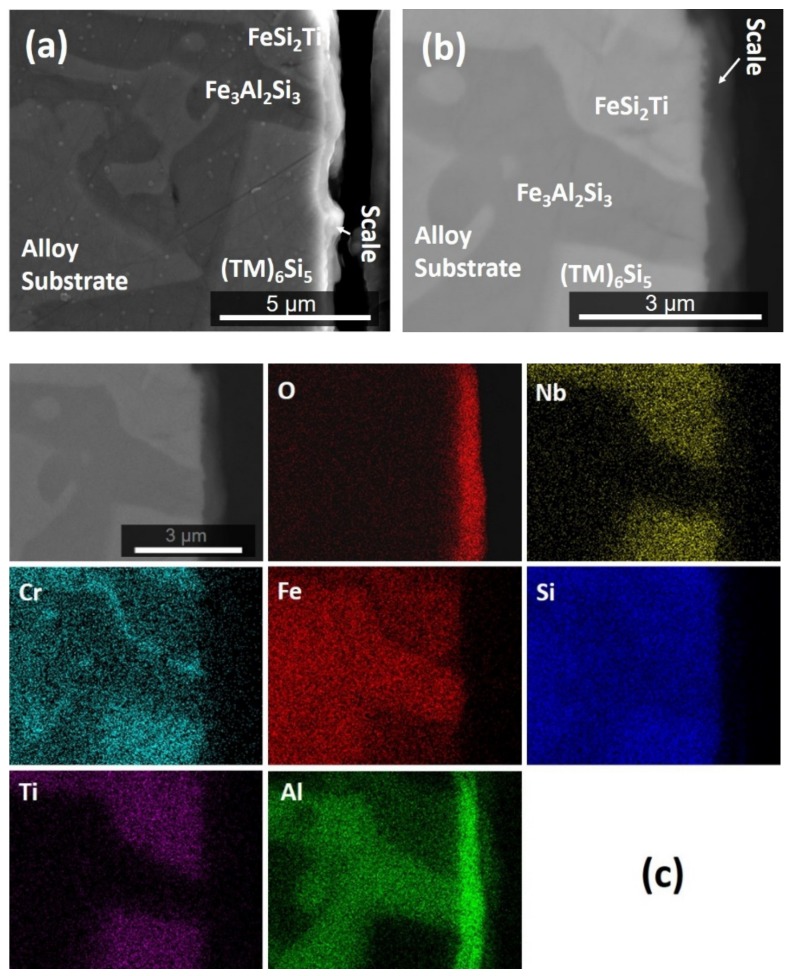
Cross-section of the alloy OHC2 after isothermal oxidation at 800 °C for 100 h: (**a**) SE image, (**b**) BSE image, (**c**) X-ray elemental maps.

The scale was continuous and adherent all over the alloy, and the oxide that formed over the TM_6_Si_5_ was thin (<1 μm). A cross-section of the oxidized specimen is shown in Figure 11. The Al_2_O_3_ blade-like whiskers that formed on the surface of the oxide over the Fe_3_Al_2_Si_3_ phase cannot be seen in this figure because they were removed during the sample preparation. According to Kofstad [34], these oxide types grow from thick films or scales and are not in direct contact with the substrate. The BSE image in the Figure 11b shows the oxide scale within the dark contrast area and a small change in the contrast of the FeSi_2_Ti phase, perhaps as a result of Al and Cr depletion. At the substrate/scale interface this phase presented some areas that suggested that oxygen had preferentially oxidized Al. The elemental distribution in the cross-section is shown in Figure 11c. Al_2_O_3_ was the main component of the scale. A continuous Al_2_O_3_ layer formed over the FeSi_2_Ti, Fe_3_Al_2_Si_3_, and TM_6_Si_5_ phases. The Fe_3_Al_2_Si_3_ phase can be observed at the substrate/scale interface and Cr enrichment at the Fe_3_Al_2_Si_3_/FeSi_2_Ti interface.

The isothermal oxidation of the alloy at 1200 °C was not studied owing to the liquation that was observed in the heat treated specimen at the same temperature.

## 5. Discussion

### 5.1. Microstructure

In the alloy OHC2-AC there was macrosegregation of all the elements and Zone A was rich in Al and Fe, with about double the Al content compared with the rest of the button. This Zone A had similar thickness with the Zone A that was formed in the alloy MG7, which was also Al-rich [7]. However, unlike the latter alloy, no aluminides were formed in the Zone A of OHC2-AC. Furthermore, in the maps in Figure 1 the latter coincided with the average composition of the alloy, with the exception of the Δχ versus δ map, unlike the Zone A in the alloy MG7, which had parameters significantly lower than the other parts of the button [7].

The solidification microstructure indicated that the TM_6_Si_5_ was the primary phase, which is in agreement with the Cr-Ti-Si and Cr-Nb-Si systems [26,29]. The TM_6_Si_5_ can be in equilibrium with TM_5_Si_3_ silicides, C40, and B20 compounds. The latter can be in equilibrium with C40 compounds [28]. The FeSi_2_Nb has the same prototype as FeSi_2_Ti and can be in equilibrium with C40 and B20 compounds [27,35]. The latter (B20) can be in equilibrium with Fe_3_Al_2_Si_3_ [30]. The available ternary phase equilibria can account for the phases observed in OHC2-AC (for the dark phase see below), OHC2-HTA, and OHC2-HTB, and for the formation of TMSi_2_ in OHC2-HTB.

The dark phase could belong to the Fe-Si-Al system, as it was essentially Nb- and Ti-free, and its Cr concentration was very low. Its composition was between those of the proposed τ_10_ and τ_11_ phases, namely τ_10_ = Al_9_Fe_4_Si_3_ (Al_57–59_Fe_24–25_Si_17–18_) and τ_11_ = Al_4_Fe_1.7_Si (Al_64–66.5_Fe_24–25_Si_9.5–11_) [32,36]. According to the crystallographic data for τ_11_, this phase was not present in the alloy OHC2. No crystallographic data is available for the τ_10_.

The (Cr,Ti)_6_Si_5_ is stable below 1565 °C [37], the FeSi_2_Ti below 1532 °C [31], and the FeSi below 1410 °C [38]. Considering the melting temperature of the FeSi, the Al addition would decrease it and Cr would be expected to slightly increase it. The Ti content would not be expected to raise the melting temperature of (Fe,Cr,Ti)(Si,Al) above 1532 °C, given that in the Si-rich region of the Fe-Ti-Si system the TiSi is stable below 1450 °C. The Al-rich Fe_3_Al_2_Si_3_ and DP phases would be expected to have a lower melting point. The melting temperatures of Nb_5_Si_3_, Ti_5_Si_3_, and Cr_5_Si_3_ are 2518, 2130, and 1780 °C, respectively, and the Fe_5_Si_3_ forms via a peritectoid reaction at 1060 °C in the binary [38] and a peritectic reaction at 1201 °C in the Fe-Ti-Si. The solution of Al in the Nb_5_Si_3_ decreases the melting temperature [39]. Fe has a strong effect on the stability of the TM_5_Si_3_. Given that the latter was rich in Ti, Cr, and Fe, it is suggested that the TM_5_Si_3_ in OHC2 was stable at temperatures lower than 1565 °C.

The TM_5_Si_3_ was present at a very low volume fraction in the form of small hexagonal grains (see Figure 2e,h) that were formed either in TM_6_Si_5_ grains or near the interface with FeSi_2_Ti or (Fe,Cr)(Si,Al). It is, therefore, possible (i) that the TM_5_Si_3_ was stable below 1532 °C, depending on its chemical composition, and (ii) that the FeSi_2_Ti and (Fe,Cr)(Si,Al) were stable below temperatures that did not differ significantly. The TM_5_Si_3_ was not observed in the Zone A, where the TM_6_Si_5_ was Fe-rich and the FeSi_2_Ti was Nb-rich. This would suggest that the formation of TM_5_Si_3_ was linked with the partitioning of Fe and Nb in the melt, and that if the melt near the TM_6_Si_5_ was starved of Fe and Nb the formation of TM_5_Si_3_ was not possible.

The microstructure in Figure 2c shows the sequence TM_6_Si_5_, FeSi_2_Ti, (Fe,Cr)(Si,Al), Fe_3_Al_2_Si_3_, and DP. The latter phases formed from the melt that surrounded the primary TM_6_Si_5_. The melt composition depended on the partitioning of solutes in TM_6_Si_5_ (Figure 4). Indeed, as the primary TM_6_Si_5_ silicide formed Fe and Al were rejected into the melt, while the other elements partitioned to the solid. Thus, the melt surrounding the TM_6_Si_5_ became richer in Fe and Al and leaner in Cr, Nb, and Ti, and from this melt the FeSi_2_Ti phase formed via the peritectic reaction L + TM_6_Si_5_ → FeSi_2_Ti. The formation of the TM_6_Si_5_, FeSi_2_Ti, and TM_5_Si_3_ starved the melt from Nb and Ti. From the Al-rich and Fe-poor melt formed the (Fe,Cr)(Si,Al), and then the Fe_3_Al_2_Si_3_ and finally the dark phase (DP).

The partitioning of Nb, Ti, Si, and Cr was opposite of that of Fe and Al in the TM_6_Si_5_. The partitioning coefficients of the elements were approximately *k*_o_^Nb^ = 2.3, *k*_o_^Si^ = 1.1, *k*_o_^Cr^ = 1.3, *k*_o_^Ti^ = 1.5, *k*_o_^Al^ = 0.14, and *k*_o_^Fe^ = 0.5. Compared with the TM_6_Si_5_ phase in the alloy OHC1, the partitioning coefficients of Fe and Cr had decreased and that of Ti had increased.

Near the bottom of the button, the volume fraction of the TM_6_Si_5_ was lower. Compared with the bulk and top of the button, the melt that solidified in these area was richer in Nb, Cr, Ti, Si, Al, and Fe. It is suggested that the melt composition had shifted close to the ternary eutectic L → Nb-rich FeSi_2_Ti + Fe_3_Al_2_Si_3_ + DPEu that was formed in this part of OHC2-AC. Compared with the alloy OHC1, the new phases in the alloy OHC2 were the TM_5_Si_3_ and Fe_3_Al_2_Si_3_ compounds. In both alloys eutectics that contained the FeSi_2_Ti phase formed in the bottom of the cast buttons, but a ternary rather than a binary eutectic was formed in the alloy OHC2.

The Ti_5_Si_3_, CrSi_2_, and Cr_5_Si_3_ silicides exhibit solubilities for third elements [37]. The solubility of Nb in Ti_5_Si_3_ and Cr_5_Si_3_ has been reported in previous studies [40,41,42,43]. Solubility of 23 at.% Nb in the Ti_5_Si_3_ has been reported in the temperature range 500 to 1200 °C, and no Nb solubility in the Cr_5_Si_3_. The solubility of Fe in Ti_5_Si_3_ is 4 at.% at 900 °C [31] and the solubility of Fe in Cr_5_Si_3_ is 4.5 at.% at 950 °C [44]. The Al solubility in Cr_5_Si_3_ is about 2.0 at.% at 800 °C [45] and in Ti_5_Si_3_ is about 12 at.% at 1000 °C [46]. The solubilities of elements that substitute Nb and Si in Nb_5_Si_3_ were discussed in a previous study [23]. The solubilities of Fe and Cr in Nb_5_Si_3_ silicide are very small [47]. In this work, the concentrations of Al and Nb in TM_5_Si_3_ were in agreement with reported solubilities, but the Fe content was higher than the reported values. The latter would suggest that in the presence of Al and Nb the solubility of Fe in TM_5_Si_3_ was increased up to 8.7 at.%.

In the Fe_3_Al_2_Si_3_ phase the Al content was in the range of 28.6 to 34.2 at.%. Considering the Al and Si contents in the Fe_3_Al_2_Si_3_ phase separately, and Cr with Fe, the composition of Fe_3_Al_2_Si_3_ was within the composition range of τ_1_ = Fe_3_Al_2_Si_3_ (Al_21.5–45_Fe_36.5–37.5_Si_8.5–41.5_) reported in a previous study [32]. The Fe_3_Al_2_Si_3_ had a limited solubility for Cr that substituted Fe, and negligible solubility for Nb and Ti. Marker et al. [36] noted the unusual combination of the broad homogeneity range of Fe_3_Al_2_Si_3_ with its very low symmetry crystal structure (triclinic). They suggested that this phase should be described as Fe_3_Al_2+*x*_Si_3−*x*_ with −0.3 < *x* < 1.3 instead of Fe_3_Al_2_Si_3_.

The TMSi_2_ had Si + Al = 65.7 at.%. According to Chen et al. [45], the solubility of Al in CrSi_2_ is up to 25 at.%, which is consistent with the Al content of TMSi_2_. It is suggested that Al enhanced the solubility of Fe and Ti in this phase, because according to Lindholm [44], the CrSi_2_ phase does not dissolve Fe.

The FeSi_2_Ti had (Si + Al) ≈ 50 at.%. After the heat treatments, its composition moved closer to the values given in a previous study [31] for the τ_1_ (FeSi_2_Ti) phase (considering Nb, Ti, and Cr together). Indeed, the compositions of the FeSi_2_Ti in the cast and heat treated conditions gave it as (Al + Si)_50–52_Fe_23–25_(Ti + Nb + Cr)_24–26_, which is consistent with the τ_1_ = FeSi_2_Ti (Si_49–50_Fe_24–25_Ti_25–26_) phase in the Ti-Fe-Si system [31]. Unlike the alloy OHC1-AC, the FeSi_2_Ti in OHC2 had up to 5.2 at.% Nb and 13.8 at.%Al, which would suggest that the presence of Al enhanced the Nb solubility in τ_1_. The Al and Cr solubilities in the τ_1_ decreased at higher temperature, which was also observed for Cr in the alloy OHC1-AC [9].

The (Fe,Cr)(Si,Al) had Si + Al about 50 at.% and high Fe solubility. The CrSi and FeSi are isostructural and have complete solubility but do not show ternary solubilities for Ti and Nb [44]. A limited Ti solubility of 1 at.% in FeSi was reported by Marker et al. [48], while Du and Shuster [37] reported negligible ternary solubilities in TiSi and CrSi. Moreover, about 2 at.% Al can be in solution in the CrSi phase according to the isothermal sections at 800 and 1100 °C of the Al-Cr-Si system [45,49]. Compared with the composition of the (Fe,Cr,Ti)Si phase in the alloy OHC1 [9], the Ti content in the (Fe,Cr)(Si,Al) in OHC2 was even lower. This phase had Cr in the range 7.9 to 9.2 at.% and up to 8.5 at.% Al. The Al content in (Fe,Cr)(Si,Al) was in good agreement with the Al solubility in FeSi (about 12 at.%) at 800 and 900 °C that was reported by Marker et al. [36,48]. After the heat treatment at 950 °C the (Fe,Cr)(Si,Al) was not stable in the alloy OHC2. The same was the case for the DP phase.

### 5.2. Oxidation

At 800 °C the oxidation of the alloy OHC2 was better compared with the alloy OHC1 [9]. It had gained less than 20% of the weight gained by OHC1 [9] and its oxidation followed parabolic kinetics after the first hour. The TGA data (Figure 7) showed consecutive very small weight changes, significantly smaller compared with the alloy OHC1 [9]. Continuous reaction with oxygen could change the microstructure of the scale with the resulting volume changes, causing some cracking in it. The alloy showed its capability to self-heal after weight loss. This was attributed to the Al addition and the oxidation of the main phases (TM)_6_Si_5_, FeSi_2_Ti, and Fe_3_Al_2_Si_3_ that formed different oxidation products in a very thin scale. There was no significant elemental depletion at the substrate/scale interface in these phases.

The oxidation was sub-parabolic with high oxidation rate in the first hour, followed by parabolic oxidation at a low rate. This behavior has been found in alumina-forming superalloys [50,51,52]. The sub-parabolic time dependence of scale growth is related to lower temperatures, and is attributed to grain-boundary-linked mechanisms (short-circuit) where fast oxygen penetration occurs, initially resulting in a high oxidation rate and then, as the oxide grows, the easy oxygen paths are blocked and the oxidation slows down [50].

As was the case in the alumina forming superalloys, the γAl_2_O_3_ and θAl_2_O_3_ were formed in the alloy OHC2. In the case of the superalloys, the αAl_2_O_3_ was formed after an extended oxidation period (more than 100 h). The transformation of γAl_2_O_3_ to θAl_2_O_3_ has been observed in the temperature range 800 to 1000 °C in NiAl alloys [53].

According to the GXRD data, the other oxides that were present in the scale together with the γAl_2_O_3_ (main oxide) and θAl_2_O_3_ were SiO_2_, Al_2_SiO_5_, FeTi_3−*x*_O*_x_*, and TiO_2_ (Figure 9). The X-ray elemental maps (Figure 11c) also confirmed the presence of Al_2_O_3_ in the scale. When γAl_2_O_3_ was found to be the main oxide on NiAl alloys at 800 °C, the diffusion mechanism was linked with its modification [53]. The other oxides formed in the scale of the alloy OHC2 could have contributed to the γAl_2_O_3_ → θAl_2_O_3_ transformation.

There is no data about the oxidation of the TM_6_Si_5_ phase with Al additions. According to this work, the low Al content in the TM_6_Si_5_ was enough to form Al_2_O_3_ over it. It is also likely that the Al_2_O_3_ formed over this phase could have different thicknesses owing to the partitioning of Al, the concentration of which was higher at the edges (Figure 4), and that the partitioning of Al could be linked with the growth of aluminum silicates, such as Al_2_SiO_5_, and SiO_2_ over the bulk of TM_6_Si_5_ (Figure 10). It is also likely that over the bulk of the TM_6_Si_5_ other oxides such as TiO_2_ had grown, allowing Si mobility towards the surface, as was discussed for the oxidation of the TM_6_Si_5_ phase in the alloy OHC1 [9], where the main oxidation products were TiO_2_, SiO_2_, and Fe_2_O_3_. Al_2_O_3_ whiskers formed on the top of the scale that developed over the Fe_3_Al_2_Si_3_. The growth of whiskers on top of the scale that grew on NiAl-based alloys was attributed to the γAl_2_O_3_ → θAl_2_O_3_ transformation [53].

Novák et al. [54] suggested that Fe-Si-Al alloys mainly form δAl_2_O_3_ with some Fe_2_O_3_ when oxidized at 800 °C, and that the Fe_2_O_3_ content in the scale decreases when the Si content in the alloys is greater than 20 at.%. In the scale of the alloy OHC2, the δAl_2_O_3_ was not confirmed by the GXRD. The Si content in Fe_3_Al_2_Si_3_ was above 30 at.% and there was some Fe enrichment at the substrate/scale interface, which could suggest the possible formation of Fe oxide(s) just below the Al_2_O_3_ scale. The GXRD did not confirm this. It is suggested that under isothermal oxidation in air at 800 °C, the Fe_3_Al_2_Si_3_ formed an Fe-oxide followed by γAl_2_O_3_, and then θAl_2_O_3_ whiskers formed at the oxide/gas interface.

In the alloy OHC1, TiO_2_ and SiO_2_ formed over the FeSi_2_Ti [9]. This phase exhibited solubility of Al in the alloy OHC2. This would explain the presence of Al_2_O_3_, Al_2_SiO_5_, and FeTi_3−*x*_O*_x_* oxides over it (Figure 9). The X-ray elemental maps in Figure 11c showed that Al_2_O_3_ was the main oxidation product. Below the areas where Al_2_O_3_ was formed there might have been some oxygen penetration. Thus, it is likely that the diffusion of oxygen in Al_2_O_3_ allowed the oxidation of Fe, Ti, and Si beneath it. Pownceby et al. [55] reported a miscibility gap between Fe_2_O_3_ and Al_2_O_3_, but they also found some solubility of Fe in Al_2_O_3_ at low temperatures. Thus, it is likely that some Fe was in solution in Al_2_O_3_. This could explain the Fe signal in the X-ray maps in Figure 10 and the absence of Fe oxide in the GXRD diffractogram (Figure 9).

## 6. Summary and Concluding Remarks 

The microstructure and isothermal oxidation at 800 °C of the silicide based alloy Si-22Fe-12Cr-12Al-10Ti-5Nb (OHC2) were studied. The cast alloy exhibited macrosegregation of all elements. The microstructures in the cast alloy and after the heat treatment at 800 °C consisted of the same phases, namely TM_6_Si_5_, TM_5_Si_3_, FeSi_2_Ti, Fe_3_Al_2_Si_3_, (Fe,Cr)(Si,Al), and an unknown phase of dark contrast (the dark phase (DP)). The latter two phases were not stable at 950 °C, where the TMSi_2_ was formed. There was evidence of endothermic reaction (s) below 1200 °C and liquation of the specimen that was heat treated at 1200 °C. The alloy followed parabolic oxidation kinetics after the first hour of isothermal oxidation at 800 °C, did not pest, and formed a self-healing scale, in which the dominant oxide was Al_2_O_3_.

The objective of this research was to find out what the effect of Al addition would be on the microstructure and oxidation of an alloy of the Si-Fe-Cr-Ti-Nb system. The alloy OHC2 was designed with the constraint and requirements that were discussed in the Section 2.

Unlike the alloy OHC1, the alloy OHC2 suffered from liquation after the heat treatment at 1200 °C. There was evidence for incipient melting in the former alloy [9] and also in the latter. On this evidence, the suitability of both alloys for BCs for Nb-silicide-based alloys is questionable. Furthermore, the experimental results of the alloy OHC2 would suggest that the high temperature stability of silicide-based Si-Fe-Cr-Ti-Nb coating alloys would be decreased by the addition of Al via alloying or interdiffusion. In our opinion, this eliminates silicide Si-Fe-Cr-Ti-Nb alloys based on the composition of OHC1 as BC components of an environmentally resistant coating for Nb-silicide-based alloys.

The data in this work and in a previous study [9] for non-pesting silicide-based Si-Fe-Cr-Ti-Nb alloys with and without Al addition would suggest that the parameters VEC and δ were important regarding the formation of SiO_2_, Cr_2_O_3_, and TiO_2_ scale or Al_2_O_3_ scale at 800 °C (see Figure 1d and compare it with the Figure 23d in the previous study [9]). Indeed, the parameter Δχ cannot differentiate between the alloys OHC1 and OHC2 (Figure 1b,c). It is suggested that the “critical” values of the alloy parameters VEC and δ for “changing” between alumina and non-alumina scales at 800 °C should be in the narrow ranges indicated, respectively, by the yellow and green color boxes in Figure 1a,b,d. Further research is needed to verify this. Additionally, Figure 1d indicates that if a multi-material BC were to be built with layers consisting of non-pesting and Al_2_O_3_ scale forming intermetallic alloys of the Si-Fe-Cr-Ti-Nb-Al and Nb-Si-Ti-Al-Hf systems, the values of the parameter δ of Nb-Si-Ti-Al-Hf intermetallic alloys that form Zone A would be in the same range as those of silicide Si-Fe-Cr-Ti-Nb alloys that do not form Al_2_O_3_ scale. We would like to suggest that VEC should be the key parameter to use to design and select non-pesting BC alloys.

The Fe-free silicide alloy OHC5 of the Si-Cr-Ti-Nb-Al system did not pest at 800 °C and formed Al_2_O_3_ scale at this temperature and at 1200 °C [9]. Being conscious of the fact that the experimental data is limited, we would like to suggest that Fe-free intermetallic alloys of the Nb-Si-Ti-Al-Hf and Si-Cr-Ti-Nb-Al systems could be components of a layered multi-material BC of an environmentally resistant coating and that their design(selection) could use the parameters δ, Δχ, and VEC, with values restricted to be in the ranges shown by the vertical and horizontal dashed lines and arrows in Figure 1.

## Figures and Tables

**Table 1:** EDS data* (at.%) of the phases in the cast alloy OHC2.

Phase	Nb (at.%)	Ti (at.%)	Cr (at.%)	Fe (at.%)	Al (at.%)	Si (at.%)
(TM)_5_Si_3_	16.2 ± 1.1	21.4 ± 1.2	16.1 ± 0.7	6.7 ± 0.9	1.8 ± 0.7	37.7 ± 1.0
	17.9–14.3	22.9–18.9	17.3–15.0	8.3–5.4	3.3–1.1	40.0–36.8
TM_6_Si_5_	7.9 ± 2.0	12.4 ± 0.8	18.5 ± 0.8	14.5 ± 2.8	2.0 ± 0.4	44.8 ± 0.4
	11.2–4.1	13.8–10.9	20.0–17.0	20.0–10.2	2.8–1.3	46.4–44.1
FeSi_2_Ti	4.3 ± 0.6	14.6 ± 0.8	5.7 ± 0.3	23.3 ± 0.4	12.5 ± 1.0	39.6 ± 0.6
	5.2–3.1	15.9–13.1	6.3–5.3	23.8–22.7	13.8–10.4	40.9–38.6
(Fe,Cr)(Si,Al)	0.1	0.4	9.2 ± 0.8	37.1 ± 0.7	8.5 ± 0.6	44.7 ± 0.4
	-	-	10.4–7.2	38.8–36.0	10.2–7.4	45.4–43.7
Fe_3_Al_2_Si_3_	0.1	0.3	4.6 ± 0.4	30.2 ± 0.6	32.2 ± 0.8	32.6 ± 0.6
	-	-	5.4–3.7	31.1–28.6	34.2–30.7	34.4–31.5
Dark phase (DP)	0.1	0.3	3.5 ± 0.3	21.3 ± 0.5	57.5 ± 1.7	17.3 ± 1.2
	-	-	4.3–2.9	22.7–20.6	59.3–54.0	19.5–16.0
Eutectic (large area analysis)	5.5±0.6	11.8 ± 0.7	5.0 ± 0.6	22.5 ± 0.7	16.0 ± 2.2	39.3 ± 1.0
	6.3–4.2	12.9–10.6	6.1–4.4	23.4–21.6	20.8–12.6	40.9–37.0
FeSi_2_Ti (in eutectic)	7.1 ± 0.8	14.4 ± 0.4	5.2 ± 0.7	21.9 ± 0.6	9.7 ± 0.8	41.7 ± 0.6
	8.5–5.8	15.5–14.0	7.6–4.2	23.0–21.0	11.6–8.5	42.5–40.0
Fe_3_Al_2_Si_3_ (in eutectic)	0.9	2.4 ± 1.9	4.5 ± 0.4	28.5 ± 1.4	29.2 ± 2.1	34.5 ± 0.7
	-	5.7–0.6	5.3–4.0	30.1–25.6	31.6–26.4	35.4–33.7
(DPEu)	2.5±0.6	5.8 ± 1.0	3.9 ± 0.3	20.7 ± 0.3	39.8 ± 3.9	27.3 ± 2.4
	3.4–1.7	7.4–4.6	4.3–3.5	21.1–20.3	45.3–35.2	30.3–23.9

Note: * data includes the average value, standard deviation, and the maximum and minimum analysis values.
